# A novel bladder cancer - specific oncolytic adenovirus by CD46 and its effect combined with cisplatin against cancer cells of CAR negative expression

**DOI:** 10.1186/s12985-017-0818-1

**Published:** 2017-08-08

**Authors:** Wenjuan Cao, Junqiang Tian, Chong Li, Yanjun Gao, Xingchen Liu, Jianzhong Lu, Yuhan Wang, Zhiping Wang, Robert S. Svatek, Ronald Rodriguez

**Affiliations:** 1 Institute of Urology, The Second Hospital of Lanzhou University, Key Laboratory of Urological Diseases in Gansu Province, Gansu Nephro-Urological Clinical Center, Cui Yingmen 82, Lanzhou, 730030 China; 2 Department of Urology, University of Texas Health Science Center San Antonio 7703 Floyd Curl Drive, San Antonio, TX 78229-3900 USA

**Keywords:** Oncolytic adenoviruses, Bladder cancer, Coxsackie virus and adenovirus receptor, Cisplatin, Apoptosis

## Abstract

**Background:**

Conditionally replicative oncolytic adenoviruses (CRAds) display significant anti-tumor effects. However, the traditional adenovirus of serotype 5 (Ad5) entering cancer cells via coxsackie virus and adenovirus receptor (CAR) can’t be utilized for bladder cancer with low expression of CAR, which limits the application of Ad5.

**Methods:**

We utilized Ad5/F11p containing the chimeric fiber gene encoding the Ad5 fiber tail domain and Ad11p fiber shaft and knob domains to construct bladder cancer-specific chimeric type viruses Ad5/F11p-PSCAE-UPII-E1A, which can infect bladder cancer cells mediated by CD46 molecule. We carried out series of experiments in vitro to research anti-tumor effect of Ad5/F11p-PSCAE-UPII-E1A and the interaction in combination with cisplatin.

**Results:**

The results demonstrated Ad5/F11p-PSCAE-UPII-E1A could infect bladder cancer cells (T24, EJ and 5637) in a CAR-independent way, and exert anti-tumor effect by blocking the cancer cells in G1 phase and inducing apoptosis. Ad5/F11p-PSCAE-UPII-E1A plus cisplatin enhanced the anti-proliferative effect and increased the number of apoptotic cells compared with viruses or cisplatin alone. Ad5/F11p-PSCAE-UPII-E1A plus cisplatin could upregulate the proteins expression of p53, Bax, and cleaved caspase-3, and downregulated Bcl-2 protein expression in T24, EJ and 5637 cells.

**Conclusion:**

We constructed a bladder cancer-specific oncolytic adenovirus and provided new combination treatment strategies for bladder cancer.

**Electronic supplementary material:**

The online version of this article (doi:10.1186/s12985-017-0818-1) contains supplementary material, which is available to authorized users.

## Background

Bladder cancer is the most common urinary tract malignant tumor [1]. The traditional treatments for bladder cancer are radical cystectomy, radiation and chemotherapy. For nonmuscle-invasive bladder cancer, the bladder tumor recurrence rate after transurethral resection of bladder tumor (TURBT) is from 50 to 70% within five years [2, 3]. Although the conventional treatments have been applied for the patients with muscle-invasive bladder cancer, half of them progress to an advanced stage with metastasis and die from this aggressive disease within 5 years [3, 4]. As a result, new effective therapeutic strategies urgently needed for the bladder cancer.

Adenoviruses are biological therapy vectors commonly used in various cancers [5, 6]. Conditionally replicative oncolytic adenoviruses (CRAds) not only lyse the cancer cells, but also release a large number of progeny virus to infect other surrounding cancer cells [7]. Thus, the infection efficiency of viruses to cancer cells is increased greatly. In recent years, CRAds made greater progress in the field of bladder tumor treatment [8–11]. The construction and advantages in anti-tumor effects of CRAds had been shown in several studies [12–14]. Furthermore, gene therapies combined with chemotherapeutic drugs could play more powerful anti-tumor effect [15, 16]. In our previous study, by using of prostate stem cell antigen enhancer (PSCAE), human bladder cancer-specific uroplakin II promoter UP II and the early adenoviral genes E1A, we constructed bladder cancer-specific oncolytic adenovirus Ad-PSCAE-UPII-E1A [17, 18]. This adenovirus is a dual specific vector which contains prostate stem cell antigen enhancer (PSCAE) and human uroplakin II (hUPII) promoter targeted bladder cancer. The hUPII promoter results in preferential expression in bladder carcinoma cells. PSCAE maintains a certain level of androgen independent activity in bladder cancer cells and can improve target gene expression in bladder cancer cells. Our previous study results suggest that combination of PSCAE with hUPII promoter is feasible in constructing bladder cancer-specific vectors [9, 17, 18]. Our oncolytic adenovirus exhibits bladder cancer-specific killing effect and exerts the synergistic antitumor effect with cisplatin on bladder cancer [9, 10, 18, 19].

The traditional oncolytic adenoviruses were always developed from adenovirus of serotype 5 (Ad5), which are endocytosis into host cells via specific coxsackie virus and adenovirus receptor (CAR) [20, 21]. However, there were low expression of CAR for some bladder cancer cells, and Ad5 can’t infect these cancer cells to exert anti-tumor effect [9, 11, 22]. To solve the above problem, we construct a new type virus called Ad5/F11p-PSCAE-UPII-E1A, which contain the chimeric fiber gene encoding the Ad5 fiber tail domain and Ad11p fiber shaft and knob domains. Codons of Ad5 fiber 579–581 aa in the polyadenylation signal were saved in pAdEasy-1/F11p. In the pAdEasy-1/F11p, the Ad11p sequence from nt 30,940 to 31,788 (Genbank accession no. AY598970), encoding 44–325 aa of Ad11p Fiber, replaced the Ad5 sequence from nt 31,169 to 32,770 (Genbank accession no. AY339865), encoding 45–578 aa of Ad5 fiber [23]. Adenovirus of serotype 11 (Ad11) depends on CD46 receptor rather than CAR to enter cancer cells. Thus, Ad11 can infect cancer cells independent of CAR expression [24–26], and Ad5/F11p had demonstrated superiority in kinds of hematopoietic cells [27].

Hence, in this study, we establish a bladder cancer-specific virus Ad5/F11p-PSCAE-UPII-E1A, which can infect CAR-positive and CAR-negative bladder cancer cells. Subsequently, we explore this virus anti-proliferative effects and influence of cell cycle aspects in bladder cancer. Finally, through in vitro experiments we demonstrated the highly improved cytolytic effect and the mechanism of new oncolytic adenovirus in combination with cisplatin by detecting apoptotic signals for bladder cancer cells.

## Methods

### Construction of Ad5/F11p-PSCAE-UPII-E1A

The fiber gene in the pAdEasy-1 was replaced with a chimeric fiber gene of Ad5/F11p (Additional file 1) encoding the Ad5 fiber tail domain and Ad11p fiber shaft and knob domains [27]. The shuttle plasmids Rp-PSCAE-UPII-E1A were digested with *Pme I* (New England Biolabs Inc., USA), and then cotransfected with backbone plasmid Ad5/F11p by electroporation in *E. coli* BJ5183 competent cells to generate the recombinant adenovirus plasmids Ad5/F11p-PSCAE-UPII-E1A by homologous recombination. Subsequently, the correct recombinant plasmids were digested with *Pac I* and transfected into HEK293 cells by Lipofectamine 2000 (Invitrogen, Carlsbad, CA, USA). The recombinant adenoviruses were identified by PCR, amplified in HEK293 cells, and purified by the routine cesium chloride density gradient centrifugation. The standard 50% tissue culture infective dose assay (TCID50) was used to quantify virus titer and then calculated the multiplicity of infection (MOI).

### Cell lines and cell culture

The cell lines used in our study contain human bladder transitional cell cancer cell lines (T24, EJ and 5637), normal human urinary cell line (SV-HUC-1), human embryonic kidney cell line (HEK293), and all of these cells were obtained from American Type Culture Collection (ATCC, Manassas, VA, USA). T24, EJ and 5637 cells were cultured in RMPI1640 medium (Invitrogen, Grand Island, NY, USA) with 10% (vol/vol) fetal bovine serum (Hyclone Laboratories). SV-HUC-1 and HEK293 cells were cultured in Dulbecco’s modified Eagle’s medium (DMEM; Invitrogen, Grand Island, NY, USA) with 10% fetal bovine serum. All cell lines used in our study were incubated in the humidified incubator under 5% carbon dioxide at 37 °C. When harvested, the cells were washed with phosphate-buffered saline (PBS), and separated with trypsin((Invitrogen, Grand Island, NY, USA).

### Polymerase chain reaction(PCR)

PSCAE gene, UPII gene, and E1A gene express in the recombinant adenovirus were identified by PCR. Firstly, harvested viruses were digested by proteinase K (Takara Biotechnology Co., Dalian, China), and then extracted virus DNA. PCR were performed according to PCR Reaction Kit (Takara) instruction. Gene expression bands were observed by agarose gel electrophoresis. The primer sequences were listed in Table 1 [9, 18].

**Table 1:** The primers used for polymerase chain reaction (PCR)

PCR primers	Primers sequence
PSCAE	Forward: 5′ GCTGACCGGTAGAGGCCAGCAGCACCCCTG 3′ Reverse: 5′ CGGTGCTAGCAACTGCTTCCGTGTGTGGCTGACAG 3’
UPII	Forward: 5’ ACT TTG AGC CTA CCC TTC C 3′ Reverse: 5′ CAG TGA GCC GAG ATT GTG 3’
E1A	Forward: 5’ CAT GCC ACA GGT CCT CAT ATA GC 3′ Reverse: 5′ GAG ACA TAT TAT CTG CCA CGG AGG 3’

*PSCAE* prostate stem cell antigen enhancer, *UPII* uroplakin II promoter, *E1A* the early adenoviral genes

### Cell viability assay

Cell Counting Kit-8 assay (CCK-8)were applied to examine cell viability. Bladder cancer cells were seeded in 96 well plates at 5000 cells per well and culture for 24 h. Ad5-PSCAE-UPII-Luc, Ad5-PSCAE-UPII-E1A and Ad5/F11p-PSCAE-UPII-E1A infected cells separately in six different MOI values. The MOI was calculated from viral particle numbers ranging from 0.01 to 1000 (0.01, 0.1 1.0, 10, 100, and 1000). After 48 h, 10 μl CCK-8(Cell Counting Kit-8, Dojindo Laboratories, Japan) was added and the absorbance was measured at wavelength of 450 nm by a multimode reader (Mithras LB 943, BERTHOLD Technologies, Germany) 4 h later. For the effect of Ad5/F11p-PSCAE-UPII-E1A combined with cisplatin, cells were infected by Ad5/F11p-PSCAE-UPII-E1A (10 MOI), and cisplatin (1 μg/ml) was then added after 24 h. The absorbance was measured after combination therapy with adenoviruses and cisplatin 24, 48, and 72 h later respectively. Each experiment was repeated three times, and each time set up six parallel well.

### Quantitative real-time PCR

The mRNA level expression of CAR and CD46 in bladder cancer cells surface were quantified by quantitative real-time PCR (qRT-PCR). The total RNA was extract using Trizol Reagent (Takara Biotechnology Co., Dalian, China), and then reversed transcribed into cDNA according to the PrimeScript RT reagent kit (Takara) by C1000 thermal cycler (Bio-Rad Laboratories, Inc., USA). Following the instructions on the SYBR Premix Ex Taq kit (Takara), the qRT-PCR was performed using the obtained cDNA in real-time quantitative instruments (CFX96, Bio-Rad Laboratories, Inc., USA). The housekeeping genes glyceraldehyde 3-phosphate dehydrogenase(GAPDH) was viewed as internal reference. Detection was repeated three times. The designs of primer sequences refer previous literature [22].

### RNA interference

To prove Ad5/F11p-PSCAE-UPII-E1A infects bladder cancer cells in a CAR-independent way, we use RNA interference technology to silence CAR expression of bladder cancer cells at post-transcriptional level. After transfected with siRNA, the expression of CAR were quantified by qRT-PCR to confirm whether or not to interfere with the expression of CAR. The bladder cancer cells were seeded in 24-well plates, and transfected with small interfering RNA (siRNA) (RiboBio Co. Ltd., Guangzhou, China) containing silencing CAR siRNA (CAR-siRNA) and negative control siRNA (NC-siRNA) by Lipofectamine 2000. The compare of CAR expression between transfected cells and untransfected cells were quantified by qRT-PCR.

### Flow cytometry analysis

Cell cycle and apoptosis were detected by flow cytometry. For cell cycle, the cells were infected with Ad5/F11p-PSCAE-UPII-E1A, Ad5-PSCAE-UPII-E1A and Ad5 -PSCAE-UPII-Luc at the MOI of 10 for 48 h separately. The cells were then fixed with 75% ethanol and suspended with the mixture of propidium iodide (BD PharMingen, San Diego, CA, USA), RNase A (Solarbio Life Sciences, Beijing, China) and Triton-X-100 (Solarbio). The cells were filtered with 300 mesh nylon meshes and measured cell cycle by flow cytometry (BD Biosciences, SanJose, CA, USA). Each experiment was repeated three times. Cell apoptosis were detected by flow cytometry to explore the interaction of Ad5/F11p-PSCAE-UPII-E1A (10 MOI) combined with cisplatin (1 μg/ml). The cells treated with combination therapy were double stained with fluorescein isothiocyanate-conjugated annexin V (annexin V-FITC; BD PharMingen) and propidium iodide (PI) and measured cell apoptosis by flow cytometry (BD Biosciences, SanJose, CA, USA).

### Western blot analysis

The expression of E1A protein, Hexon protein, and apoptosis-related proteins (p53, Bcl-2, Bax, cleaved caspase-3, and caspase-3) were detected by Western blot analysis. The total proteins were extracted using RIPA with phenylmethanesulfonyl fluoride (PMSF) (Beyotime Biotechnology, Jiangsu, China), and quantized using a BCA Protein Assay Kit (Beyotime). Sodium dodecyl sulfatepolyacrylamide gel electrophoresis (SDS-PAGE) was carried out to separate the total protein. The separated protein was transferred to a PVDF membrane (Millipore, Bioprocess Technology Center, Billerica, MA, USA), blocked with skimmed milk powder, and incubated in the first antibodies overnight at 4 °C. The membranes were incubated in horseradish peroxidase -conjugated secondary antibodies for 2 h. After extensive washing with 20 ml Tris-buffered saline-Tween (TBST; Solarbio), the proteinwas visualized at Fast Chemiluminescence Image System (ImageQuant 350, GE Healthcare Life Sciences, UAS). The housekeeping genes β-actin was viewed as internal reference.

### Statistical analysis

Statistical software SPSS 13.0 (SPSS Inc., Chicago, IL, USA) was used to analyze the study results. The results were expressed by the average value with standard deviation (X ± SD). Analysis of variance (ANOVA) and the two-tailed t test were applied to evaluate the discrepancy between groups. *P*-values <0.05 (*) and *P*-values <0.01 (**) mean statistically difference and statistically significant difference between groups respectively.

## Results

### Construction of Ad5/F11p-PSCAE-UPII-E1A

The recombinant adenovirus plasmids Ad5/F11p-PSCAE-UPII-E1A (Ad5F11p-E1A) were produced by homologous recombination between Rp-PSCAE-UPII-E1A and Ad5/F11p. The control viruses in our study were Ad5-PSCAE-UPII-E1A (Ad5-E1A) and Ad5-PSCAE-UPII-Luc (Ad5-Luc). When constructed, PSCAE gene, UPII gene, and E1A gene express in Ad5/F11p-PSCAE-UPII-E1A was identified by PCR. In the Additional file 2, the lanes 1 and 5 are bands of marker, the lanes 2, 3 and 4 are gene bands of PSCAE, UPII, and E1A of Ad5/F11p respectively, and the lanes 6, 7 and 8 are gene bands of PSCAE, UPII, and E1A of Ad5 respectively. The molecular sizes of marker are 100 bp, 200 bp, 300 bp, 400 bp, 500 bp, 700 bp, and 1000 bp respectively (from the bottom up). The PCR results showed that PSCA enhancer,human UPII promoter and E1A were 327 bp, 314 bp, and 541 bp respectively, which meant viral constructs were suitable and it can be used for follow-up study (Suppl 2).

### CAR and CD46 expression in bladder cancer cells

**Fig. 1:**
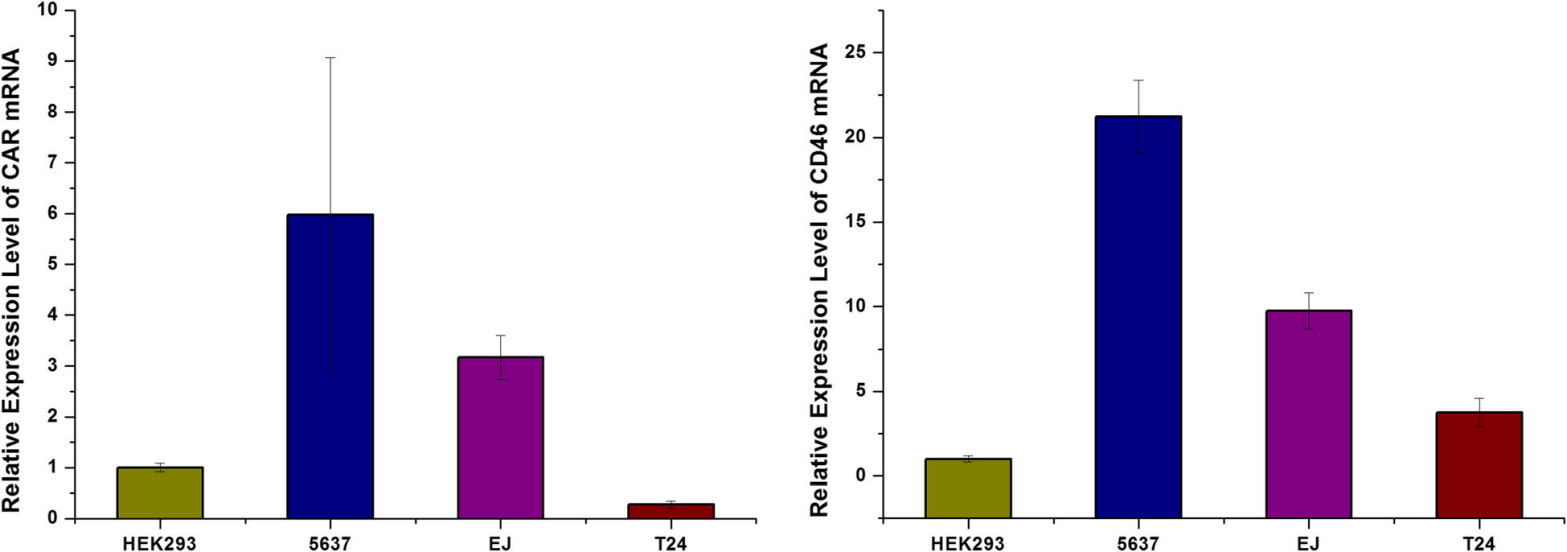
The mRNA expression level of CAR and CD46 receptors in bladder cancer cells. The mRNA level express of CAR and CD46 in bladder cancer cells 5637, EJ, and T24 were quantified by qRT-PCR, and the control are HEK293 cells. The two-tailed t test were applied to evaluate the discrepancy between groups. *P*-values <0.01 (**) mean statistically significant difference between groups

**Fig. 2:**
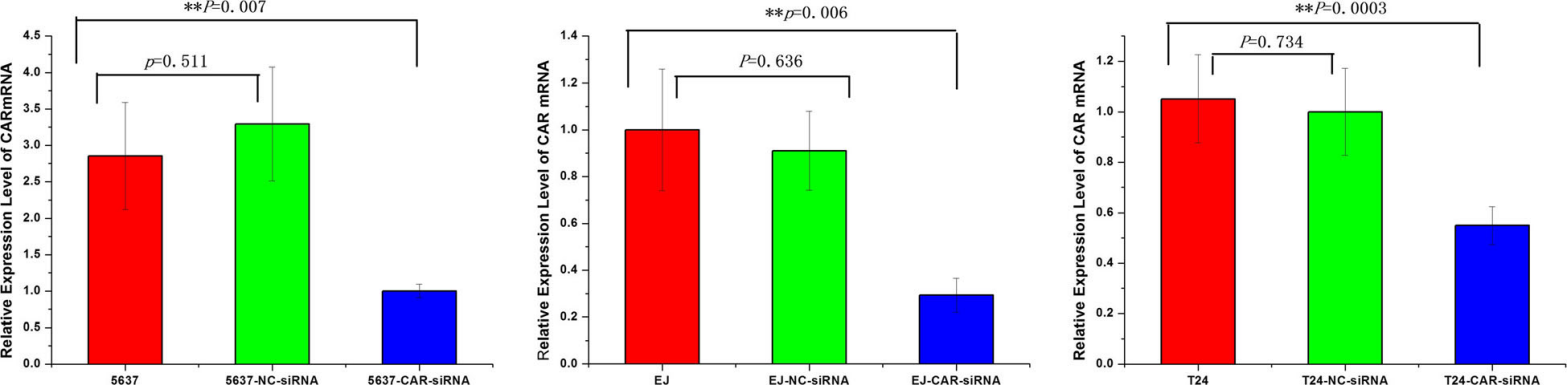
The compare of CAR expression between transfected cells and untransfected cells. The compare of CAR expression between transfected cells and untransfected cells were quantified by qRT-PCR. For 5637, EJ, and T24 cells, the mRNA level express of CAR were no different between untransfected cells and transfected with negative control siRNA (NC-siRNA) cells, but there were statistically significant difference between untransfected cells and transfected with silencing CAR siRNA (CAR-siRNA) cells (**p < 0.01)

In order to prove Ad5/F11p-PSCAE-UPII-E1A enters into bladder cancer by CD46 molecules but not CAR for killing bladder cancer cells specifically, the mRNA level expression of CAR and CD46 in bladder cancer cells surface were quantified by qRT-PCR. HEK 293 was used as control cells. The two-tailed t test were applied to evaluate the discrepancy between groups. Expression of CAR in 5637 cells was the highest, followed by EJ cells, while expression in T24 was the lowest compared with the HEK 293(all *p* < 0.01). CD46 gene expression was higher in bladder cancer cells than in HEK 293 (all *p* < 0.01) (Fig. 1). After transfected with CAR siRNA, the expression of CAR between untransfected cells and transfected cells with CAR-siRNA were quantified by qRT-PCR. The results indicated the expression of CAR between untransfected cells and transfected cells with CAR-siRNA had statistically significant difference (all *p* < 0.01). But the expression of CAR between untransfected cells and transfected cells with NC-siRNA had no statistically difference (all *p*>0.05) (Fig. 2).

### Anti proliferative effect of Ad5/F11p-PSCAE-UPII-E1A for bladder cancer cells

**Fig. 3:**
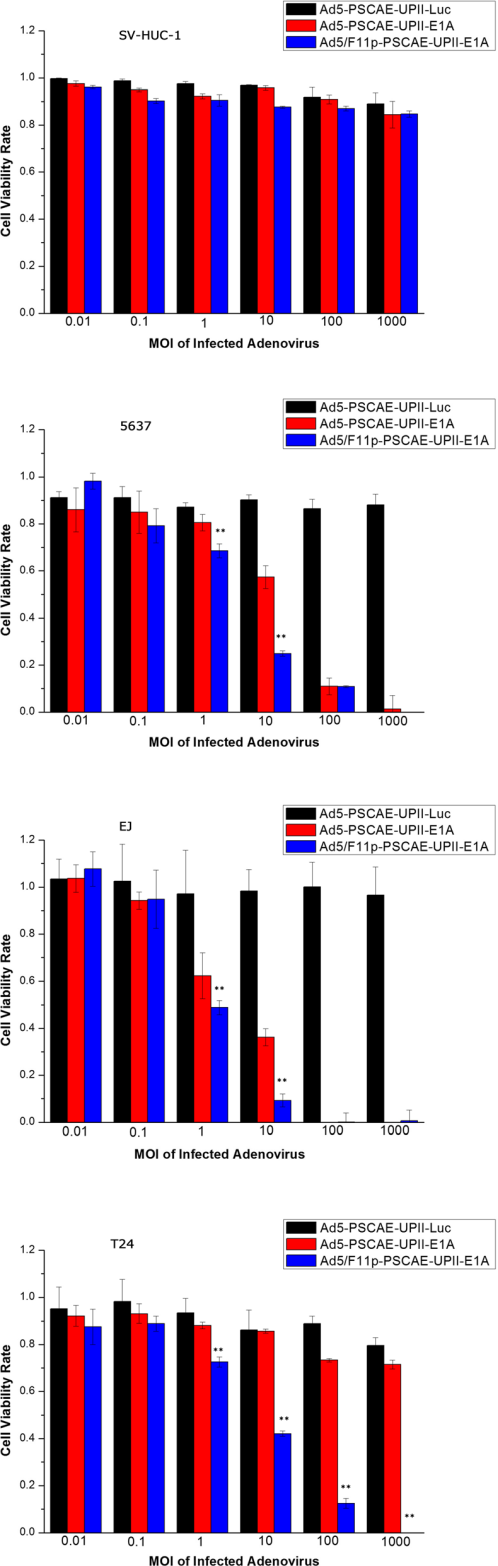
The cytotoxic effects of Ad5/F11p on bladder cancer cells. The cytotoxic effects of this three virusesin in six incremental MOI at 0.01, 0.1, 1.0, 10, 100, and 1000 were assessed by CCK-8 assay. The average value of three independent experiments with standard deviation were used to express the data results. The cytotoxic effects of Ad5-PSCAE-UPII-E1A and Ad5/F11p-PSCAE-UPII-E1A in six incremental MOI were compared on 5637, EJ, and T24 cells (**p < 0.01 means statistically significant difference)

We used CCK-8 assay to compare the cytotoxic effects of Ad5-PSCAE-UPII-Luc, Ad5-PSCAE-UPII-E1A and Ad5/F11p-PSCAE-UPII-E1A in six incremental MOI at 0.01, 0.1, 1.0, 10, 100, and 1000. Ad5/F11p-E1A play a more powerful anti-proliferative effect than Ad5-E1A at the MOI of 1.0 and 10 for EJ and 5637 cells with statistically significant difference (all *p* < 0.01). The anti-proliferation effect of Ad5/F11p-E1A in T24 cells was much enhanced compared with Ad5-E1A at the MOI of 1.0, 10, 100, 1000(all *p* < 0.01). Simultaneously, Ad5- Luc, Ad5- E1A and Ad5/F11p-E1A had no cytotoxic effects for normal human urinary cell line SV-HUC-1 (Fig. 3).

### The expression of E1A protein and Hexon protein in bladder cancer cells infected with Ad5F11p- E1A

**Fig. 4:**
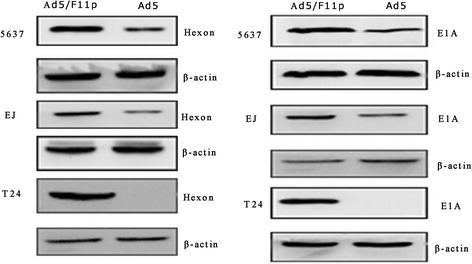
The expression of Hexon and E1A in bladder cancer cells. Ad5/F11p-PSCAE-UPII-E1A (10 MOI) and Ad5-PSCAE-UPII-E1A (10 MOI) were used to infect 5637, EJ, and T24 cells, and the differential expression of Hexon protein and E1A protein were detected by Western blot analysis after 48 h. The housekeeping genesβ-actin was viewed as internal reference. Ad5/F11p represented Ad5/F11p-PSCAE-UPII-E1A and Ad5 represented Ad5-PSCAE-UPII-E1A

The expression of E1A protein and Hexon protein in bladder cancer cells after being infected with Ad5/F11p-PSCAE-UPII-E1A or Ad5-PSCAE-UPII-E1A were detected by Western blot analysis. More E1A protein and Hexon protein can be detected in EJ and 5637 cells infected with Ad5F11p- E1A than these two cells infected with Ad5- E1A. E1A protein and Hexon protein can not be detected in T24 cells infected with Ad5- E1A, but can be detected in T24 cells infected with Ad5F11p- E1A (Fig. 4). This suggests Ad5F11p- E1A could infect bladder cancer cells (T24, EJ and 5637) in a CAR-independent way.

### The effect of Ad5F11p- E1A for different bladder cancer cells with or without CAR gene expression

**Fig. 5:**
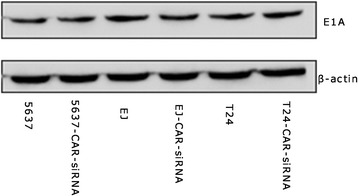
The comparison of E1A protein expression between untransfected cells and transfected with siRNA cells. Ad5/F11p-PSCAE-UPII-E1A (10 MOI) were used to infect untransfected and transfected with silencing CAR siRNA (CAR-siRNA) bladder cancer cells(d), and compare the E1A protein expression by Western blot analysis after 48 h. The housekeeping genesβ-actin was viewed as internal reference

**Fig. 6:**
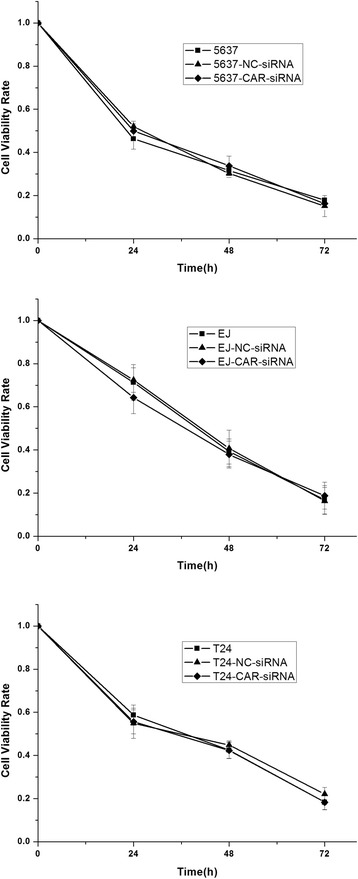
The comparison of cytotoxic effects between untransfected cells and transfected with siRNA cells. Ad5/F11p-PSCAE-UPII-E1A (10 MOI) were used to infect 5637, EJ, and T24 cells which were treated with CAR-siRNA, NC-RNA and untreated with siRNA respectively, and then assess Ad5/F11p-PSCAE-UPII-E1A cytotoxic effects for untransfected and transfected cells at different points in time by CCK-8 assay

We carried out RNA interference to silence CAR gene expression of bladder cancer cells, and then infected these cells with Ad5/F11p-PSCAE-UPII-E1A. We compared the replication and anti-proliferative effect of Ad5F11p- E1A in bladder cancer cells with or without CAR gene expression. There is no difference between expression of E1A protein in CAR positive and CAR silenced bladder cancer cells (Fig. 5). The CCK-8 assay showed Ad5F11p- E1A played the same anti-proliferative effect in 5637, EJ, and T24 cells treated with CAR-siRNA, NC-RNA and untreated with siRNA at different points in time (Fig. 6).

### The impact of Ad5F11p- E1A on the cell cycle

**Fig. 7:**
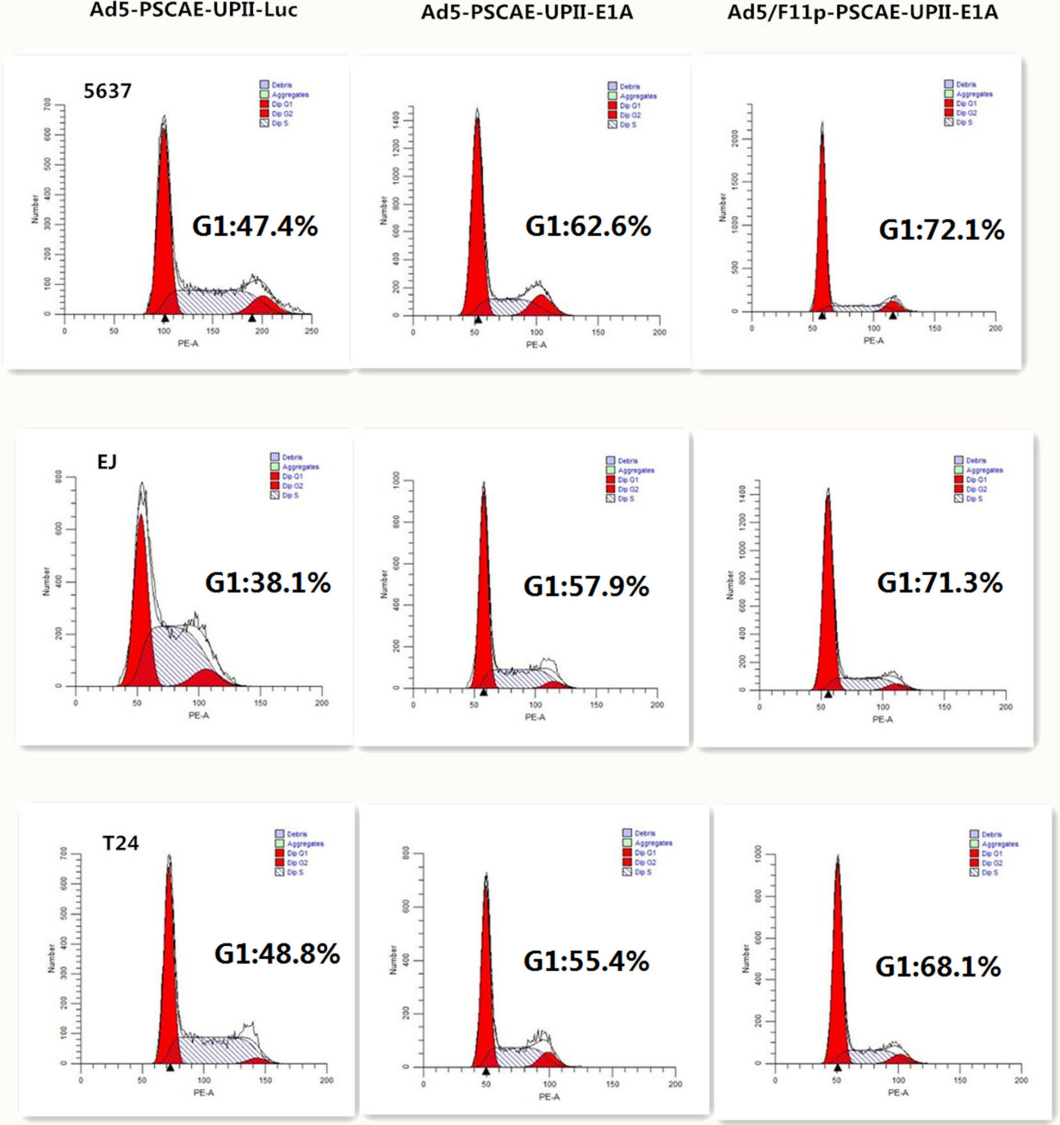
Cell cycle analysis on bladder cancer cells infected with Ad5/F11p. The bladder cancer cells were infected with Ad5/F11p-PSCAE-UPII-E1A, Ad5-PSCAE-UPII-E1A or Ad5-PSCAE-UPII-Luc at a MOI of 10 for 48 h, and Cell cycle were detected by flow cytometry. The bladder cancer cells were infected with Ad5-PSCAE-UPII-Luc as a control

We explored cytotoxic mechanism of Ad5/F11p-PSCAE-UPII-E1A to bladder cancer by cell cycle analysis. T24, EJ, and 5637 cells were infected with Ad5/F11p-PSCAE-UPII-E1A, Ad5-PSCAE-UPII-E1A, and Ad5-PSCAE-UPII-Luc at the MOI of 10 respectively. After stained with propidium iodide, cell cycles were analyzed by flow cytometry. The proportion of G1 phase rate in T24, EJ, and 5637 cells infected with Ad5F11p-E1A was 68.1 ± 0.9%, 71.3 ± 1.3%, and 72.1 ± 0.4% respectively. The proportion in T24, EJ, and 5637 cells infected with Ad5-E1A in G1 phase was 55.4 ± 0.8%, 57.9 ± 1.7%, and 62.6 ± 0.6% respectively (Table 2, Fig. 7).

**Table 2:** The cells rate arrested in G1 phase (X ± SD) (%)

Cell lines	Ad5-PSCAE-UPII-Luc	Ad5-PSCAE-UPII-E1A	Ad5/F11p-PSCAE-UPII-E1A
5637	47.4 ± 1.5	62.6 ± 0.6	72.1 ± 0.4
EJ	38.1 ± 2.7	57.9 ± 1.7	71.3 ± 1.3
T24	48.8 ± 0.3	55.4 ± 0.8	68.1 ± 0.9

*X ± SD* average value ± standard deviation

### The anti-proliferative effect of Ad5F11p-E1A combined with cisplatin

**Fig. 8:**
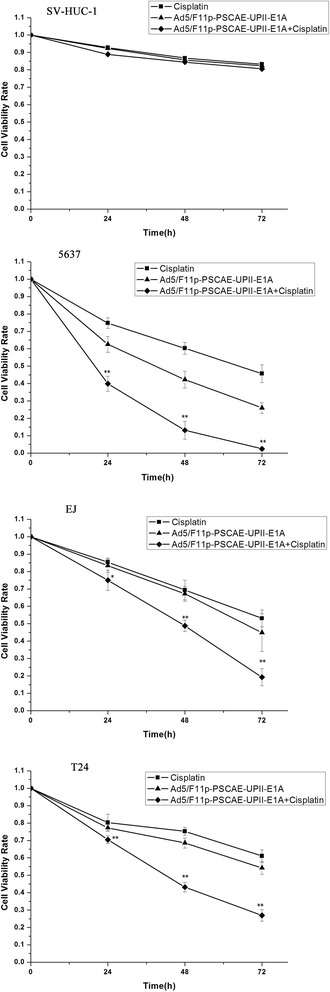
The effects of Ad5/F11p combined with cisplatin on bladder cancer cells. The interaction effects of Ad5/F11p-PSCAE-UPII-E1A (10 MOI) combined with cisplatin (1 μg/ml) at different time points (24, 48, and 72 h after the combined effects) were assessed by CCK-8 assay. The mean of three independent experiments with standard deviation were used to express the data results. The cytotoxic effects of Ad5/F11p-PSCAE-UPII-E1A alon and the interaction effects of Ad5/F11p-PSCAE-UPII-E1A combined with cisplatin were compared on 5637, EJ, and T24 cells (*p < 0.05 means statistically difference, and **p < 0.01 means statistically significant difference)

We observed the anti-proliferative effect of Ad5/F11p-PSCAE-UPII-E1A combinated with cisplatin on bladder cancer cells at different time points (24, 48, and 72 h after the combined effects) by CCK-8 assay. Ad5F11p-E1A (10 MOI) combined with cisplatin (1 μg/ml) had killed more cells than Ad5F11p-E1A(10 MOI) alone in T24 and 5637 cells at all different time points with statistically significant difference (all *p* < 0.01). For EJ cells, the combination also showed stronger killing effect than viruses alone (*p* < 0.05 on day 1, and *p* < 0.01 on day 2 and 3). Ad5F11p-E1A (10 MOI) alone, cisplatin(1 μg/ml) alone, and the combination had no obviously cytotoxic effects for normal human urinary cell line SV-HUC-1 at different time points (Fig. 8).

### Ad5F11p-E1A improves apoptosis

**Fig. 9:**
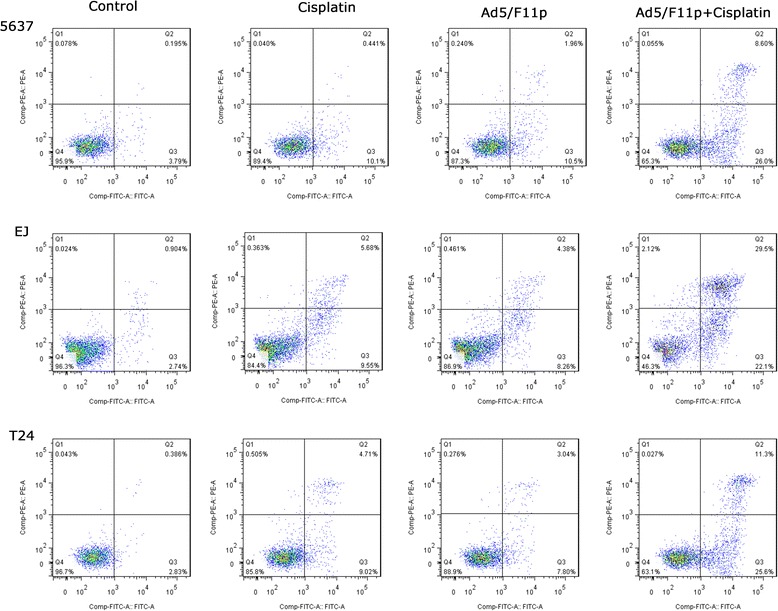
Cell apoptosis analysis on bladder cancer cells treated with cisplatin and Ad5/F11p. Cell apoptosis were detected by flow cytometry to explore the interaction of Ad5/F11p-PSCAE-UPII-E1A combined with cisplatin. The 5637, EJ, and T24 bladder cancer cells were treated with Ad5/F11p (10 MOI) alone, cisplatin (1 μg/ml) alone, or Ad5/F11p (10 MOI) plus cisplatin (1 μg/ml) respectively for 48 h, incubated with fluorescein isothiocyanate-conjugated annexin V (AV-FITC) and propidium iodide (PI) for 15 min, and then detect cell apoptosis by flow cytometry. The bladder cancer cells were untreated with cisplatin, Ad5/F11p, and Ad5/F11p combined with cisplatin as a control

Next, we probed the interaction mechanism of Ad5/F11p-PSCAE-UPII-E1A(10 MOI) combinated with cisplatin (1 μg/ml) for bladder cancer by cell apoptosis analysis. For T24 cells, the rate of early apoptotic cells in four different groups (control group, cisplatin(1 μg/ml) alone group, Ad5F11p-E1A (10 MOI) alone group, and Ad5F11p-E1A (10 MOI) + cisplatin(1 μg/ml) group) were 2.83%, 9.02%, 7.80%, 25.6% respectively. For 5637 and EJ cells, the early apoptotic cells proportion of were also gradually increased in this four different groups. The percentage of early apoptosis was increased significantly in combination of Ad5F11p-E1A with cisplatin compared with Ad5F11p-E1A or cisplatin alone in bladder cancer cells (Fig. 9).

### The apoptosis-related proteins expression by western blot analysis

**Fig. 10:**
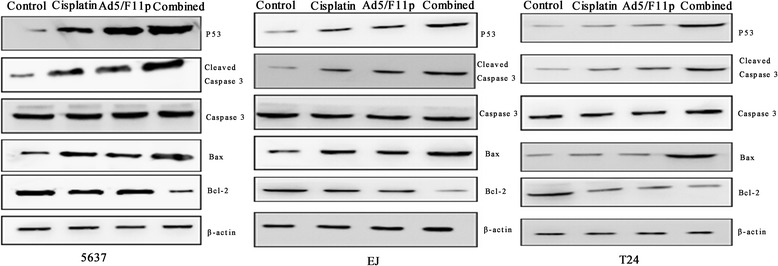
The apoptosis-related proteins expression on bladder cancer cells treated with cisplatin and Ad5/F11p. The 5637, EJ, and T24cells bladder cancer cells were treated with Ad5/F11p-PSCAE-UPII-E1A (10 MOI) alone, cisplatin (1 μg/ml) alone, Ad5/F11p-PSCAE-UPII-E1A (10 MOI) plus cisplatin (1 μg/ml), or PBS for 48 h respectively, and then assessed the expression of apoptosis-related proteins by Western blot analysis. The housekeeping genesβ-actin was viewed as internal reference. Ad5/F11p represented Ad5/F11p-PSCAE-UPII-E1A and combined represented Ad5/F11p-PSCAE-UPII-E1A plus cisplatin.

In order to further illustrate the inducing early apoptosis mechanism of the combination effect, we detected apoptosis-related proteins (p53, Bcl-2, Bax, cleaved caspase-3, and caspase-3) expression by Western blot analysis. The western blot results showed p53, Bax, and cleaved caspase-3 protein expression were increased significantly in bladder cancer cells treated with Ad5F11p-E1A or cisplatin than the untreated control cells. Whereas, Bcl-2 protein expression were decreased in these treated cells. The bladder cancer cells treated with Ad5F11p-E1A plus cisplatin expressed higher proteins of p53, Bax, and cleaved caspase-3 than the cells treated with Ad5F11p- E1A or cisplatin alone. Conversely, Bcl-2 protein expression were lower in the combination group comparing with the Ad5F11p-E1A or cisplatin alone group (Fig. 10).

## Discussion

Adenoviruses are biological therapy vectors commonly used in various cancers [5, 6]. Due to the particular mode of tumor destruction, oncolytic adenoviruses can increase the sensitivity of bladder cancer cells for chemotherapy and radiotherapy [28, 29]. In addition, the special anatomical structure of bladder offers convenient conditions for oncolytic adenoviruses infusion therapy, which can not only decrease the adverse effects caused by systemic therapy, but also greatly reduces the immune system to the removal of viruses [30, 31].

Adenovirus of serotype 5 (Ad5) were most traditional gene therapy vectors which entered cancer cells with CAR receptor to kill cancer cells [20, 21]. The previous studies have reported the expression of CAR in the surface of bladder cancer cells [11, 22], but low expression of CAR in T24 cells was observed in our experiment compared with HEK293 control cells, which limits the application of Ad5. The previous study had showed that histone deacetylase inhibitor could increase the expression of CAR and the replication of adenovirus, and furthermore enhance viruses killing to cancer cells [32]. However, histone deacetylase inhibitor could only affect the role of tumor specific promoter, and it was not effective for all oncolytic adenovirus. In order to extend Ad5 application for CAR negative expression bladder cancer we construct bladder cancer-specific chimeric type viruses Ad5/F11p-PSCAE-UPII-E1A according to replace the fiber gene of pAdEasy-1 with Ad5/F11p concluding Ad5 fiber tail domain and Ad11p fiber shaft and knob domains. The advantage of this new chimeric virus were that it can infect bladder cancer cells mediated by CD46 molecule [11, 27, 33, 34]. CD46 is the identified receptor for B and partial D subgroup adenoviruses and expressed in the majority of cell surface [35]. Ad11 has dual tropism for both CD46 and desmoglein 2 (DSG-2). Desmoglein-2 as the primary high-affinity receptor could triggers events for transient opening of intercellular junctions, and thus improves access to CD46 receptors [36]. Our results showed Ad5/F11p-PSCAE-UPII-E1A was effective for bladder cancer cells and its infectivity were not correlated with the expression quantity of CAR in bladder cancer cells surface, which expanded the application of viral therapy in the treatment of bladder cancer.

The anti-tumor mechanism of oncolytic adenovirus is still not entirely clear. The latest research suggested the expression of cell cycle gene were related with development, staging and classification of urothelial carcinoma [37], and oncolytic adenovirus might inhibit the growth of tumor cells by inducing cell apoptosis [38]. Our results showed that more bladder cancer cells were arrested in G1 phrase with significant difference after being infected with Ad5/F11p-PSCAE-UPII-E1A. This may be related with the produce of E1A protein inducing apoptosis. E1A is an anti-cancer gene which can block the cell cycle in G1 phase and induce apoptosis [9, 38]. We speculate Ad5/F11p-PSCAE-UPII-E1A exerted anti-tumor effect by blocking the cancer cells in G1 phase and inducing apoptosis in bladder cancer.

Although Ad5/F11p-PSCAE-UPII-E1A had demonstrated anti-tumor effect in our studies, virus therapy alone for bladder cancer was still insufficient. Therefore, we investigated the combination effect of Ad5/F11p-PSCAE-UPII-E1A with cisplatin. Cisplatin is a very broad clinical application chemotherapy drug, and also is one of most commonly used drugs in bladder cancer chemotherapy [15, 39]. With the extensive application of cisplatin in clinical, the side effects are noticed [16, 40]. Along with the progress of disease and the long-term use of cisplatin, many tumors become resistant to cisplatin in patients [41]. In recent years, some scholars had proved the synergistic effect in combination therapy of adenoviruses and cisplatin for a variety of tumor cells in order to lesson cisplatin side effects and drug resistance [15, 42, 43]. Our data showed the combined effects of the viruses with cisplatin in inhibiting cells proliferation and inducing apoptosis for bladder cancer. The combined effects improve the biological treatment level of oncolytic adenovirus for bladder cancer.

Although our studies had indicated effectiveness of Ad5/F11p-PSCAE-UPII-E1A for bladder cancer and effects combined with cisplatin, there is still much to be further investigated. Bladder cancer animal model should be established to investigate the combination therapy of adenovirus Ad5/F11p-PSCAE-UPII-E1A and radiation therapy or chemotherapy in vivo studies.

## Conclusions

In general, we successfully constructed out of a new virus chimeric type virus Ad5/F11p-PSCAE-UPII-E1A. This viruses exerted powerful anti-tumor effect in vitro for bladder cancer of CAR negative expression and provided an advantageous combination therapy with cisplatin for bladder cancer, which extended the range of gene therapy.

## Additional files

Additional file 1: The basic structure chart of skeleton plasmid Ad5/F11p. According to cutting into pAdEasy-1 gene sequence by restriction enzyme EcoRI and overlap extending fiber gene of Ad5 and Ad11p by polymerase chain reaction method, the fiber gene in the pAdEasy-1 was replaced with a chimeric fiber gene of Ad5/F11p encoding the Ad5 fiber tail domain and Ad11p fiber shaft and knob domains. The yellow arrow area represent the chimeric Ad5/F11p fiber. (TIFF 356 kb)

Additional file 2: The identified results of Ad5/F11p-PSCAE-UPII-E1A by PCR method. PSCAE gene, UPII gene, and E1A gene express in the recombinant adenovirus of Ad5/F11p-PSCAE-UPII-E1A and the control adenovirus Ad5-PSCAE-UPII-E1A were identified by PCR method. Gene expression bands were observed in agarose gel electrophoresis. The lanes 1 and 5 are bands of marker. The lanes 2, 3 and 4 are gene bands of PSCAE, UPII, and E1A of Ad5/F11p respectively, and the lanes 6, 7 and 8 are gene bands of PSCAE, UPII, and E1A of Ad5 respectively. The molecular sizes of marker are 100 bp, 200 bp, 300 bp, 400 bp, 500 bp, 700 bp, and 1000 bp respectively (from the bottom up). The molecular sizes of PSCAE gene, UPII gene, and E1A gene are 327 bp, 314 bp, and 541 bp respectively. (TIFF 17684 kb)

## Abbreviations

ANOVA: Analysis of variance
CAR: Coxsackie virus and adenovirus receptor
CCK-8: Cell Counting Kit-8 assay
CRAds: Conditionally replicative oncolytic adenoviruses
CY 3: the cyanine three
E1A: Early adenoviral genes
GAPDH: The housekeeping genes glyceraldehyde 3-phosphate dehydrogenase
MOI: Multiplicity of infection
PCR: Polymerase chain reaction
PI: propidium iodide
PMSF: Phenylmethanesulfonyl fluoride
PSCAE: Prostate stem cell antigen enhancer
qRT-PCR: quantitative real-time PCR
SDS-PAGE: Sodium dodecyl sulfatepolyacrylamide gel electrophoresis
TBST: Tris-buffered saline-Tween
TCID50: The standard 50% tissue culture infective dose assay
TURBT: Transurethral resection of bladder tumor
UP II: Uroplakin II promoter
