# Supplementary figures and images for: A novel bladder cancer - specific oncolytic adenovirus by CD46 and its effect combined with cisplatin against cancer cells of CAR negative expression
Source: Virol J. 2017 Aug 8;14:149. doi: 10.1186/s12985-017-0818-1 (PMC5549334; doi:10.1186/s12985-017-0818-1)

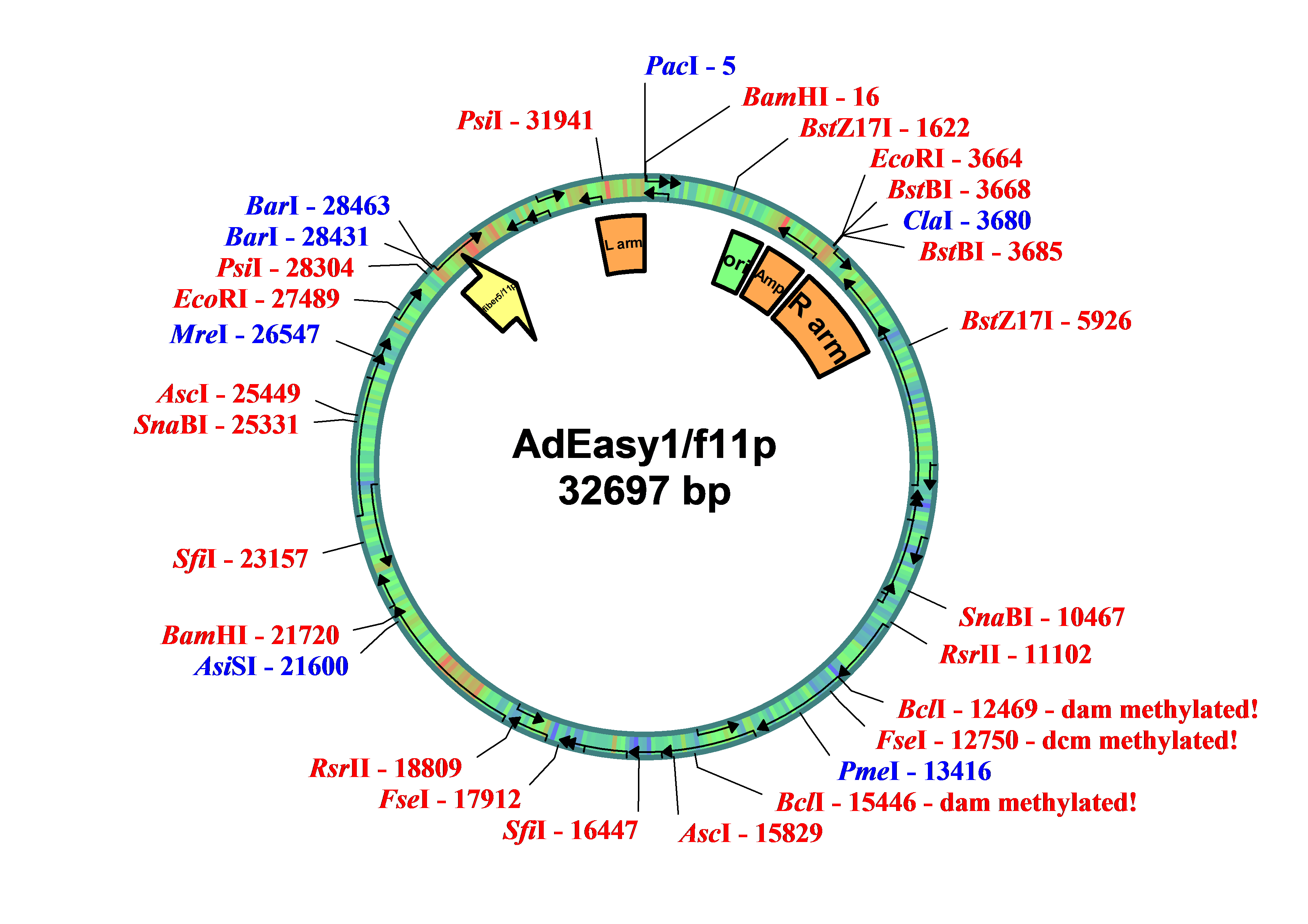

Supplement: Supplementary file 1 — The basic structure chart of skeleton plasmid Ad5/F11p. According to cutting into pAdEasy-1 gene sequence by restriction enzyme EcoRI and overlap extending fiber gene of Ad5 and Ad11p by polymerase chain reaction method, the fiber gene in the pAdEasy-1 was replaced with a chimeric fiber gene of Ad5/F11p encoding the Ad5 fiber tail domain and Ad11p fiber shaft and knob domains. The yellow arrow area represent the chimeric Ad5/F11p fiber. (TIFF 356 kb) [file 12985_2017_818_MOESM1_ESM.tif]

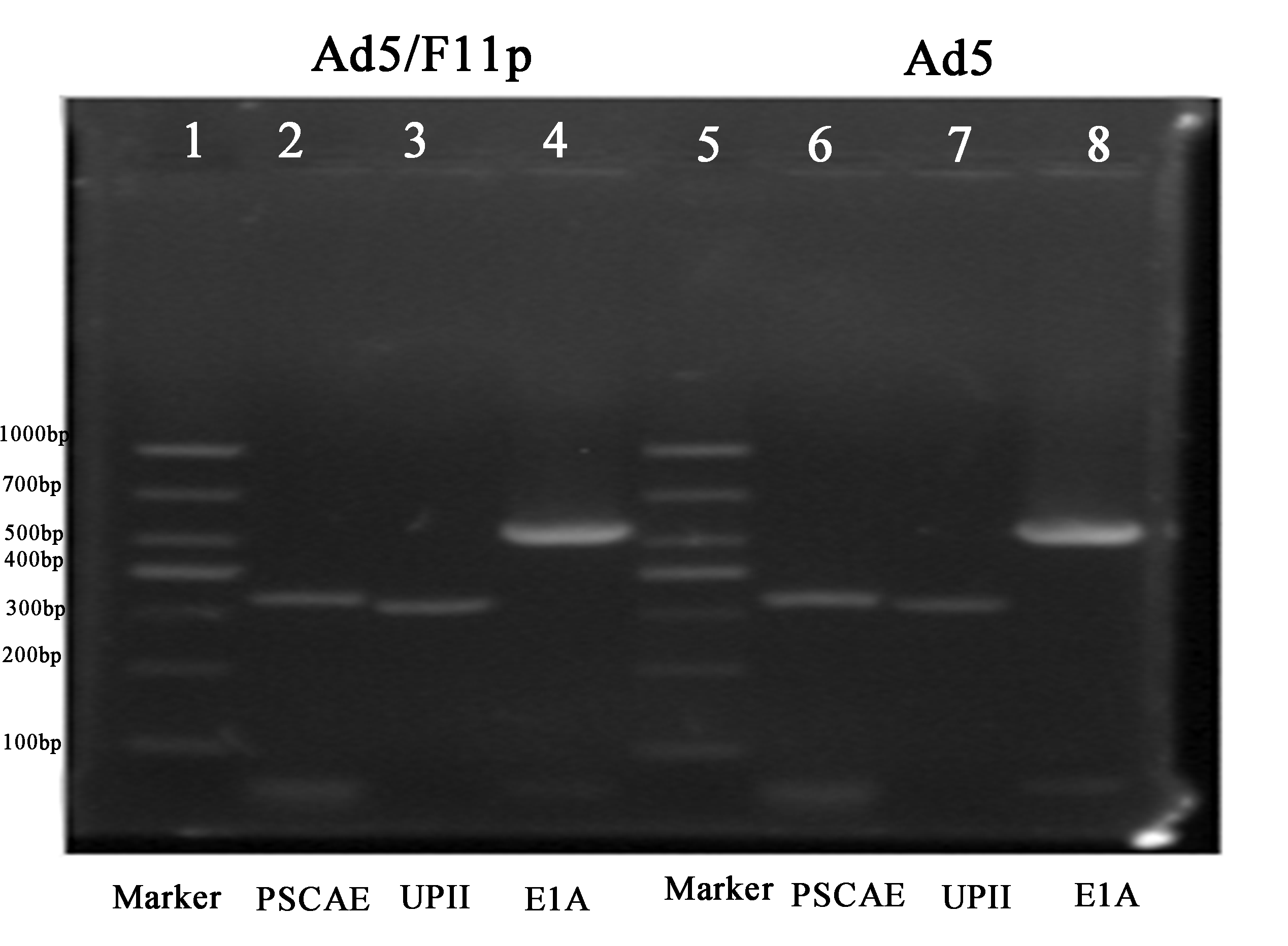

Supplement: Supplementary file 2 — The identified results of Ad5/F11p-PSCAE-UPII-E1A by PCR method. PSCAE gene, UPII gene, and E1A gene express in the recombinant adenovirus of Ad5/F11p-PSCAE-UPII-E1A and the control adenovirus Ad5-PSCAE-UPII-E1A were identified by PCR method. Gene expression bands were observed in agarose gel electrophoresis. The lanes 1 and 5 are bands of marker. The lanes 2, 3 and 4 are gene bands of PSCAE, UPII, and E1A of Ad5/F11p respectively, and the lanes 6, 7 and 8 are gene bands of PSCAE, UPII, and E1A of Ad5 respectively. The molecular sizes of marker are 100 bp, 200 bp, 300 bp, 400 bp, 500 bp, 700 bp, and 1000 bp respectively (from the bottom up). The molecular sizes of PSCAE gene, UPII gene, and E1A gene are 327 bp, 314 bp, and 541 bp respectively. (TIFF 17684 kb) [file 12985_2017_818_MOESM2_ESM.tif]
